# Agricultural-Grade Apple Cider Vinegar Is Remarkably Attractive to *Drosophila suzukii* (Diptera: Drosophiliadae) in Mexico

**DOI:** 10.3390/insects11070448

**Published:** 2020-07-15

**Authors:** Rodrigo Lasa, Saide Aguas-Lanzagorta, Trevor Williams

**Affiliations:** Instituto de Ecología AC, Xalapa, Veracruz 91073, Mexico; saideaguas13@gmail.com

**Keywords:** spotted wing drosophila, apple flavored vinegar, apple nectar, Droskidrink, Suzukii Trap, raspberry, polytunnel production

## Abstract

Due to its availability and low cost, apple cider vinegar (ACV) is a frequently used as an attractant for monitoring the invasive spotted wing drosophila, *Drosophila suzukii*. In laboratory cage experiments, the attraction of ACV alone was compared with ACV in mixtures with different concentrations of acetic acid, propionic acid, different hydrolyzed proteins, synthetic fruit flavors (strawberry, blackberry and apple) and the addition of fruit nectars (grape, pineapple and apple). The addition of 5% apple nectar to ACV significantly increased fly captures, whereas other combinations were similar to or less attractive than ACV alone. Apple flavored vinegar was not attractive to flies. Captures did not vary significantly among the brands of ACV commonly sold in Mexico, except for one poorly-performing brand, but cup traps baited with an agricultural-grade ACV unfit for human consumption captured approximately two-fold more flies than the commercial attractants Suzukii Trap, Suzukii Trap Max Captures or ACV alone in cage experiments. Field trials performed in polytunnels planted with raspberry crops in Mexico resulted in two-fold to ten-fold higher numbers of *D. suzukii* captured by the agricultural-grade ACV compared to Droskidrink (a mixture of ACV, red wine and sugar), Suzukii Trap, Suzukii Trap Max Captures or edible grade ACV alone. The species selectivity of the agricultural grade ACV was similar to that of other attractants tested. Agricultural-grade ACV also captured higher numbers of female than male flies in field trials. We conclude that the remarkably high attractiveness and low cost of agricultural-grade ACV makes it a useful tool for monitoring *D. suzukii* populations in berry crops.

## 1. Introduction

The spotted wing drosophila, *Drosophila suzukii* (Matsumura) (Diptera: Drosophilidae), is an invasive pest that has recently become widely distributed in Europe and the Americas [1,2]. This fly was first detected in Mexico in Michoacán State in 2011 and subsequently spread rapidly to all berry-producing regions of the country [3]. It is capable of attacking a wide range of thin-skinned fruits [4,5,6], due to the serrated ovipositor that allows females to lay eggs in fruits that are still attached to the crop [7]. Raspberry and blackberry are highly preferred fruits for this pest [8], although many other species of fruit crops are also attacked by this fly [9]. The economic impact and regulatory export trade requirements have led growers to continuously monitor the presence of this pest in the field. Insecticides such as spinosad and spinetoram are applied frequently to control this pest during the berry production period.

Monitoring traps can help to define pest distribution, record seasonal activity and contribute to evaluating the success of appropriate control schemes. Several trap designs and attractants have been evaluated to monitor *D. suzukii* since this pest invaded Europe and the Americas [10,11]. Recent studies have evaluated the performance of vinegars, such as apple cider vinegar [10,12,13,14,15,16,17] and brown rice vinegar [18,19], wine [20], fermenting yeasts [13,21,22], different mixtures of yeast, wine and vinegar [20,23,24,25,26], artificial mixtures of volatile compounds produced naturally during fermentation [8,27,28,29], plant essential oils in mixtures with raspberry juice [30], and commercial attractants [31,32,33], for the monitoring of this pest.

The principal berry-growing region in Mexico is located in the state of Michoacán. As elsewhere [12,14,15,16,17], apple cider vinegar (ACV) is the most commonly used attractant in Mexico and is recommended by Mexico’s Ministry of Agriculture [34]. This is because ACV is easy to find, relatively cheap, is transparent, allowing trapped flies to be readily visualized with the naked eye, and is effective at attracting *D. suzukii* adults. Mexican berry growers favor the use of ACV because it is a stable attractant that requires little maintenance and can be left in traps for periods of several weeks without loss of attractiveness to flies.

Previous evaluations by us revealed that ACV performed as well as, or better than, two commercial attractants sold in Mexico, Suzukii Trap^®^ (Bioiberica, Barcelona, Spain; a mixture of organic acids and peptides) and Z-Kinol^®^ (a mixture of kairomones based on fruit volatiles), or a home-made mixture of raspberry pulp and sucrose commonly used by Mexican berry growers [35]. In contrast, fermenting yeast + sucrose solution alone, or mixed with ACV + ethanol in a two-component trap device, resulted in significantly higher numbers of captured flies than standard ACV baited traps [25]. The attraction of this pest is also influenced by yeast species and growth media [36,37]. Despite the high attraction of fermenting yeasts, their use among growers in Mexico has not been widespread because fermentation and rate of degradation tend to vary, whereas ACV is a uniform, stable product that is simple to handle in the field. A variety of ACV brands and apple flavored vinegars are sold in Mexico for culinary purposes, whereas unprocessed agricultural-grade ACV, not fit for human consumption, is sold as an organic herbicide or for the cleaning of agricultural irrigation systems.

Others have noticed that the combination of ACV with other substances can improve attraction, presumably because mixtures of volatile components interact positively with the pest’s olfactory system. For example, the addition of wine to ACV [20,23], or wine mixed with sugar [38], improved the capture of *D. suzukii* when compared with ACV alone. Similarly, although the commercial attractant Suzukii Trap includes a mixture of peptides and organic acids, the addition of hydrolyzed proteins to ACV has not been tested to our knowledge. Moreover, the addition of synthetic fruit essences to a sugar–yeast mixture improved the attraction of *D. suzukii* females [39], whereas fly attraction to ACV mixed with raspberry fruit essence was not increased compared to ACV alone [35]. Other synthetic fruit essences and fruit juices have yet to be tested in mixtures with ACV. The fermentation source of different vinegars can also influence the attraction of *D. suzukii*, at least in the case of brown rice vinegar [18].

In this study we examined the attraction of *D. suzukii* to ACV in mixtures with small quantities of organic acids, proteins, synthetic fruit essences and fruit nectars. We also compared fly attraction to commercially available vinegars and ACV-based attractants currently available in Mexico. Finally, polytunnel experiments were performed to confirm the high attraction of *D. suzukii* to an unprocessed agricultural-grade ACV, not suitable for human consumption, which proved to be more attractive than any other product tested to date.

## 2. Materials and Methods

### 2.1. Insect Colony

A laboratory colony of *D. suzukii* was started in the Instituto de Ecología AC, Xalapa, Veracruz, with adults obtained from naturally infested wild blackberry, *Rubus fruticosus* L., collected at Xico, Veracruz (19°25′59′′ N; 97°1′58′′ W, 1385 m altitude) in June 2015. Adults were allowed to oviposit in a cornmeal-based artificial diet [40], dispensed into 300 mL plastic cups and covered with a fine nylon gauze under laboratory conditions: 24 ± 1 °C, 60 ± 10% relative humidity (RH) and 12:12 h light–dark (L:D) photoperiod. Adult females and males were collected every day and kept together in cages until required for experiments.

### 2.2. Evaluation of Apple-Cider-Vinegar-Based Attractants

Pairwise comparisons were performed to evaluate the capture of *D. suzukii* with ACV alone or ACV in several different mixtures. Experiments were performed using “La Costeña” (Ecatepec, Mexico) brand ACV as the reference treatment because this brand consistently proved attractive to *D. suzukii* in previous experiments [25].

For experiments, small traps were constructed from 120 mL plastic cups (35 mm diameter, 87 mm height) that were perforated on the lateral wall, with three equidistant holes through which translucent conical tubes (9 mm external diameter, 6 mm internal diameter, 20 mm length) were inserted to decrease the frequency of fly escape once inside the trap. Holes were placed 45 mm above the base and the lower half of the cup was covered with cream-colored paper to avoid fly responses to different colored vinegars, as described previously [37]. A 50 mL volume of ACV or ACV + additives were placed in each trap with 5 µL Tween 80 (Golden Bell, Mexico City, Mexico) to reduce the surface tension of the ACV that acted as a drowning solution. Traps were placed 20 cm apart at the opposite sides of home-made acrylic cages (30 × 30 × 30 cm), ventilated with a 26 × 26 cm piece of 0.3 mm nylon mesh on the lateral walls and roof of the cage, but not on the front face. Traps were initially assigned to random positions at the sides of each cage but subsequently changed position for each new replicate. A moist cotton wool wick was placed at the center of the cage as a water source for the duration of each experiment. To reduce the influence of the surroundings, each cage was surrounded by a white background and illuminated with fluorescent tubes providing 3500 lux of incident light at the top of the cage. Forty non-starved flies of 5-d-old (20 females and 20 males) were released inside the cage under laboratory conditions at 24 ± 1 °C, 60 ± 10% RH and 12:12 h (L:D) photoperiod in the same laboratory as the insect colony. Each experimental cage was placed at least 50 cm away from other cages. Trapped flies were collected, sorted by sex and counted from traps 23 h later. The remaining flies inside the cage were collected and discarded. Four independent cages were evaluated simultaneously. Each test was performed twice (with the traps rotated at each position) to give a total of eight replicates per experiment. Each group of flies was tested once and then discarded. According to this methodology, the following six different sets of experiments were performed:

*Experiment 1.* The first experiment compared the attraction of La Costeña ACV with the commercial lure Suzukii Trap (Bioiberica, Barcelona, Spain). This experiment was performed to ensure that the reference ACV treatment had a similar attraction under caged conditions as the commercial lure, as previously observed in the field [35];

*Experiment 2.* To examine whether diluted vinegar performed better than undiluted vinegar, we compared the attraction of La Costeña ACV (undiluted) with dilutions of 40, 60 or 80% La Costeña ACV (*v*/*v*) in unchlorinated water;

*Experiment 3.* To examine whether the addition of organic acids to ACV improved attraction, two tests were performed to compare fly attraction to La Costeña ACV alone or in mixtures with 5% (*v*/*v*) acetic acid (Sigma Aldrich, St. Louis, MO, USA; >99% purity) or 2.5% (*v*/*v*) propionic acid (Sigma Aldrich, >99% purity);

*Experiment 4.* To examine whether mixtures of ACV and hydrolyzed proteins increased fly attraction, the captures of La Costeña ACV alone were compared with those of ACV in mixtures with four different hydrolyzed proteins commonly used for trapping tephritid fruit flies: (i) CeraTrap (Bioiberica, Barcelona, Spain), an enzymatic hydrolyzed protein of animal origin, (ii) Captor 300 (Promotora Agropecuaria Universal, Mexico City, Mexico; comprising 33% hydrolyzed proteins), (iii) Flyral (Bioiberica, Barcelona, Spain; 36% hydrolyzed proteins) and (iv) Winner 360 (Internacional Química de Cobre, Mexico City, Mexico; 35.5% hydrolyzed proteins). These three last hydrolyzed proteins came from acidic hydrolysis of undefined plant proteins. In all cases, proteins were present at a concentration of 8% (*v*/*v*);

*Experiment 5.* To determine whether the use of synthetic fruit aromas increased the attraction to ACV, experiments compared the captures of La Costeña ACV alone with those of ACV in mixtures with 0.1% of a synthetic fruit essence (apple, strawberry or blackberry) (Saborex, Querétaro, Mexico). This concentration was selected for testing as previous tests indicated that higher concentrations of a synthetic essence of raspberry proved repellent to *D. suzukii* adults [35];

*Experiment 6.* To examine potential favorable interactions of ACV with fruit juice aromas, an experiment was performed to compare captures of La Costeña ACV alone with ACV mixed with 5% (*v*/*v*) of one of three fruit nectars, sweetened products made from concentrated fruit juices: grape, apple or pineapple (Grupo Jumex, Mexico City, Mexico). An additional test compared La Costeña ACV alone with ACV + 15% (*v*/*v*) apple nectar, as the initial test with ACV + 5% apple nectar resulted in high numbers of captured flies.

### 2.3. Laboratory Cage Attraction of D. suzukii to Vinegars and Other Attractants

The capture of *D. suzukii* flies in traps baited with several types of vinegars was evaluated under laboratory cage conditions in six independent tests (named Experiments A–F). All tests were performed using the same methodology, in which the capture of *D. suzukii* flies in traps baited with one of four attractants was evaluated simultaneously in two nylon net cages (with exception of Experiment F that was performed using four cages set up simultaneously). Cages were 60 × 60 × 90 cm and contained a single potted blackberry plant (80 cm high) placed at the center of the cage. Cages were placed 1 m apart on a table in a controlled environment room at 26 ± 1 °C temperature, 65 ± 10% RH and 12:12 h (L:D) photoperiod. A trap design based on that of Carroll (2016), comprising a red opaque polyethylene cup (460 mL capacity; Primo Cuevas, Ecatepec, Mexico), was modified by the addition of a black strip of electrical tape and 20 lateral holes of 3.2 mm diameter uniformly distributed in two rows (10 equidistant holes per row) at 2/3 of the height of the cup, as described previously [25]. The cup was sealed by a transparent flat lid. This opaque red and black cup was used in order to reduce the possible effect of the attractant color that varied among the products that were to be tested. Traps baited with 150 mL of each attractant were hung at a height of 70 cm at each of the four corners of the cage, 45–50 cm apart. A 10 µL volume of Tween 80 was added to all attractants to reduce the surface tension of the liquid drowning solution, with the exception of Suzukii Trap and Suzukii Trap Max Captures that already appeared to contain tensioactive agents.

In Experiments A–C, 20 females and 20 males of *D. suzukii*, aged between 4 and 6 days, that had not been deprived of food, were released into the center of each cage using an entomological aspirator. In order to achieve higher numbers of captured flies, in Experiments D–F, 40 females and 40 males of similar aged flies, not previously deprived of food, were released in each cage. At 23 h after release, captured flies were sorted by sex and counted. The remaining flies inside the cage were collected and discarded. The cups containing each of the four attractants were assigned randomly to the corners of each cage. Each trap was rotated on four occasions after each 23 h experimental period for each of the two cages to give eight replicates in total. Attractants were evaluated in the following six Experiments (A–F) using different vinegars. The composition of each of the commercial vinegars is given in the Supplemental Material (Table S1).

*Experiment A.* To compare attraction of different types of vinegars, the following four types of vinegars were evaluated: (i) La Costeña ACV used as the reference treatment in Experiments 1–6, (ii) rice vinegar (Sugoi, Guadalajara, Mexico), (iii) red wine vinegar (Carbonell, Córdoba, Spain) and (iv) sugarcane vinegar (Clemente Jacques, Ecatepec, Mexico).

*Experiment B*. Following favorable results for La Costeña ACV in Experiment A, a similar test was performed to compare the following four ACV brands that are common in Mexican supermarkets: (i) La Costeña ACV reference treatment, (ii) “Clemente Jacques” ACV (Sabormex, Ecatepec, Mexico), (iii) “Barrilito” ACV (Grupo Dimex, Ecatepec, Mexico) and (iv) “Heinz” ultra-filtered organic ACV (Heinz, San Pedro Tlaquepaque, Jalisco, Mexico).

*Experiment C.* To compare the efficacy of La Costeña ACV with apple-flavored vinegars (AFV) that comprise a mixture of sugarcane vinegar, colorants and flavors, four products were compared: (i) the reference La Costeña ACV, (ii) “La Anita” AFV (Grupo La Anita, Mérida, Mexico), (iii) “San Miguel” AFV (San Miguel, Xalapa, Mexico) and (iv) “La Faburrita” AFV (La Faburrita, Xalapa, Mexico).

*Experiment D.* To compare the efficacy of La Costeña ACV with other grades of ACV four products were compared: (i) La Costeña ACV reference treatment (ii) “Ruiz” ACV, a brand selected for its low price (~40% cheaper than the reference ACV) (Hermanos Ruiz, Xalapa, Mexico), (iii) “Picolargo” human edible grade ACV (Frutícola Picolargo, Ciudad Guerrero, Mexico) and (iv) “Picolargo” agricultural-grade ACV, considered as unfit for human consumption (Frutícola Picolargo, Ciudad Guerrero, Mexico). Both formulations of Picolargo ACV were selected following informal comments received from berry growers in the northwest of Mexico.

*Experiment E.* Following favorable results for Picolargo agricultural grade ACV in Experiment D, we compared attraction among four different ACV-based attractants that had been reported to be highly attractive to *D. suzukii* in previous studies by ourselves and others: (i) La Costeña ACV reference treatment (ii) La Costeña ACV + 5% (*v*/*v*) apple nectar, (iii) “Droskidrink”, a mixture of 75% La Costeña ACV, 25% red wine (“California” brand, Valle Redondo, Naucalpan de Juárez, Mexico) and 20 g/L raw brown sugar, an attractant used for *D. suzukii* in Europe and the United States [13,38] and, (iv) Picolargo agricultural-grade ACV.

*Experiment F.* A final experiment was performed to compare the attraction of Picolargo agricultural-grade ACV with a commercial attractant widely sold in Mexico, Suzukii Trap and a new formulation of this attractant named Suzukii Trap Max Captures. Four products were compared: (i) La Costeña ACV reference treatment, (ii) Suzukii Trap, (iii) Suzukii Trap Max Captures, (iv) Picolargo agricultural-grade ACV.

### 2.4. Attraction to Agricultural-Grade ACV in Polytunnel Raspberry Crops

Given the high attraction in laboratory studies and the low cost of agricultural-grade ACV, two independent polytunnel experiments were performed in raspberry (var. Adelita) in March–April 2019 and April 2020 to compare the agricultural-grade ACV with other popular attractants used for *D. suzukii*. The first experiment was performed at the “Los Lobos” farm (19°52′31′′ N; 102°20′34′′ W, 1985 m altitude), whereas the second experiment was performed at the “El Sauz” farm (20°2′9′′ N; 102°11′34′′ W, 1591 m altitude). Both farms were close to the town of Zamora, Michoacán State, Mexico. Climatic conditions during the field trials were typical for March and April in this region (mean temperature: 18–21 °C, maximum 28–32 °C, minimum 8.5–11 °C, mean monthly precipitation 3.5–5.0 mm). Both experiments were performed in open-sided polytunnels covered with white polythene, 6.6 m in width, 4 m in height and 50–65 m in length, divided into six blocks of ~0.25 ha/block. Each tunnel covered three rows of raspberry plants. One-liter colorless transparent cup traps were used, each containing 250 mL of one of three different attractants. Each replicate comprised one trap in one experimental block of raspberry plants (six in total). The attractants evaluated in the first experiment were: (i) La Costeña ACV reference treatment, (ii) Picolargo agricultural-grade ACV and, (iii) Droskidrink (described in laboratory Experiment E). In the second experiment, the following attractants were evaluated: (i) Picolargo agricultural-grade ACV, (ii) Suzukii Trap and, (iii) Suzukii Trap Max Captures. Droskidrink and all ACV-based attractants were mixed with 10 µL of Tween 80 to improve fly drowning, but this detergent was not used for Suzukii Trap or Suzukii Trap Max Captures that already contained tensioactive agents. Traps were placed among raspberry plants on metal stakes in a line at 1.5 m above the ground, at a distance of 10–12 m between traps and at least 10 m from the edge of the polytunnel. Experimental blocks (n = 6) were separated by 15–20 m. Traps containing attractants were randomly assigned to stakes within each block. Traps were inspected at 7-day intervals. At inspection, captured flies were placed in 70% ethanol and traps were rotated clockwise by one position for each new sample during three consecutive weeks of the study (one sample at each position within the block). The attractants were not renewed during the study. Samples in ethanol were taken to the laboratory, where all drosophilids were counted and *D. suzukii* individuals were identified and counted by sex.

### 2.5. Statistical Analysis

Pairwise comparisons of mean numbers of *D. suzukii* flies trapped in the La Costeña ACV reference treatment, and the other attractants in laboratory caged Experiments 1–6, were performed by paired *t*-tests. The numbers of captured *D. suzukii* flies in caged Experiments (A–F) involving four attractants were √*x* + 0.5 transformed to stabilize variance and subjected to two-way ANOVA with attractant and fly sex as factors. For polytunnel trials, the numbers of *D. suzukii* flies and other drosophilids captured in each trap were averaged over the three-week trial period and mean values (n = 6 per treatment) were analyzed by fitting generalized linear models (GLMs) with a negative binomial distribution in a randomized block design, followed by a Bonferroni test for comparison of treatment means. The *D. suzukii* females captured and attractant specificity, measured as the ratio of *D. suzukii* flies divided by the total number of drosophilids captured per trap, were used to fit GLMs with a Poisson distribution with overdispersion, followed by Bonferroni mean comparisons. All analyses were performed using the R-based program Jamovi v.1.1.9.0 [41].

## 3. Results

### 3.1. Evaluation of Apple Cider Vinegar-Based Attractants

The results of Experiments 1–6 are presented in Table 1. The number of flies captured in traps baited with La Costeña ACV was similar to that of the commercial product Suzukii Trap (Experiment 1), indicating that La Costeña ACV is highly attractive to *D. suzukii* (Table 1). The dilution of La Costeña ACV in water tended to reduce its attraction to flies, although this difference became statistically significant only in the most dilute treatment (40%) in Experiment 2. Marked reductions in attraction to traps baited with ACV + 5% acetic acid or ACV + 2.5% propionic acid were observed compared to the La Costeña ACV reference treatment in Experiment 3 (Table 1). The addition of different types of hydrolyzed proteins (Captor, Winner, Flyral or CeraTrap) to ACV in Experiment 4 did not significantly affect numbers of flies captured in any treatment. Similarly, none of the synthetic fruit flavors (blackberry, strawberry, apple) mixed with ACV significantly affected attraction by flies (Experiment 5), compared to La Costeña ACV alone. Finally, the addition of 5% apple nectar to ACV significantly increased attraction over that of La Costeña ACV alone, but no such effects were observed in mixtures of ACV with the same concentration of grape or pineapple nectars (Experiment 6). The addition of 15% apple nectar to ACV resulted in a numerical increase in fly captures, but did not differ significantly from that of La Costeña ACV alone (Experiment 6) (Table 1).

### 3.2. Laboratory Cage Attraction of D. suzukii to Vinegars and Other Attractants

**Figure 1:**
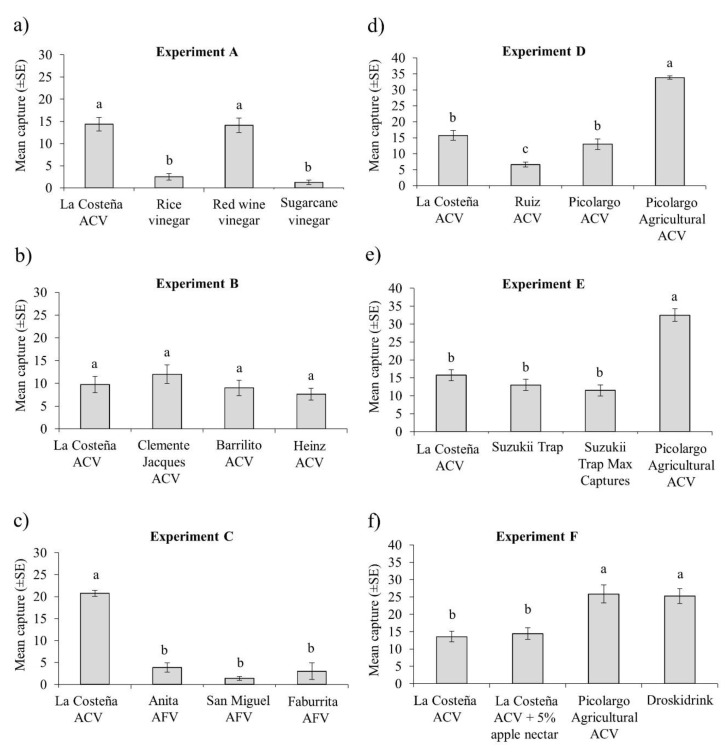
Mean number of *D. suzukii* flies captured per trap in laboratory cage Experiments (A–F) with (**a**) La Costeña apple cider vinegar (ACV) and other types of vinegars, (**b**) different brands of ACV, (**c**) different brands of apple flavored vinegar (AFV), (**d**) different brands of ACV including an agricultural-grade ACV unfit for human consumption, (**e**) comparison of agricultural-grade ACV with two commercial attractants, (**f**) comparison of agricultural-grade ACV and ACV + 5% apple nectar with Droskidrink (ACV, red wine and sugar). La Costeña ACV was included as a reference treatment in all experiments. Vertical bars indicate SE. Columns headed by different letters differ significantly (ANOVA, Tukey *p* < 0.05).

The results of the caged tests on attraction to vinegars are presented in Figure 1. Fly attraction to La Costeña ACV and red wine vinegar was similar and was significantly higher than attraction to either rice vinegar or sugarcane vinegar (F = 47.13, df = 3, 56, *p* < 0.001) (Experiment A; Figure 1a). Overall, 48% more males than females were captured in this experiment (F = 6.84; df = 1, 56; *p* = 0.011), but the interaction of vinegar*sex was not significant (F = 1.63; df = 3, 56; *p* = 0.192).

Traps baited with different brands of ACV (La Costeña, Clemente Jacques, Barrilito, Heinz) in Experiment B (Figure 1b), captured similar numbers of flies in all cases (F = 1.62, df = 3, 56, *p* = 0.193), independent of sex (F = 0.025; df = 1, 56, *p* = 0.875), or the interaction of brand*sex (F = 0.033; df = 3, 56, *p* = 0.992). On average, 51 ± 17% (mean ± SD) of captured flies were females across all treatments.

Traps baited with any of the apple-flavored vinegars (AFV, Anita, San Miguel or Faburrita) in Experiment C (Figure 1c), captured significantly fewer flies than the La Costeña ACV treatment (F = 65.07, df = 3, 56, *p* < 0.001), independent of sex (F = 0.505; df = 1, 56, *p* = 0.480), and with no significant interaction of vinegar type*sex (F = 1.853; df = 3, 56, *p* = 0.148) (Figure 1c). On average, 63 ± 14% (mean ± SD) of captured flies were females across all treatments.

Comparison of different brands of ACVs with Picolargo agricultural-grade ACV (Experiment D) revealed that agricultural-grade ACV captured significantly higher numbers of flies than any of the other ACVs tested (F = 48.86, df = 3, 56, *p* < 0.001), independent of sex (F = 0.668; df = 1, 56, *p* = 0.417), although the mean capture of females for agricultural ACV (57%) was significantly higher than for the human edible grade ACV (44–45%) (attractant*sex interaction F = 2.870; df = 3, 56, *p* = 0.044). Ruiz brand ACV was the least attractive vinegar, whereas La Costeña and Picolargo (human edible grade) ACVs captured intermediate numbers of flies (Figure 1d). On average, 48 ± 18% (mean ± SD) of captured flies were females across all treatments.

The significantly higher capture of flies by agricultural-grade Picolargo ACV was also observed in Experiment E (Figure 1e), in which this product was compared to La Costeña ACV, Suzukii Trap or the re-formulated product Suzukii Trap Max Captures (F = 37.77, df = 3, 56, *p* < 0.001), independent of sex (F = 0.198; df = 1, 56, *P* = 0.658), or the interaction of attractant*sex (F = 0.181; df = 3, 56, *p* = 0.909). On average, 51 ± 9% (mean ± SD) of captured flies were females across all treatments.

A comparison of the most attractive products and mixtures identified in the previous experiments with Droskidrink (a mixture of ACV, wine and sugar), frequently used in Europe and the United States (Experiment F), revealed that Picolargo agricultural-grade ACV captured similar numbers of flies as Droskidrink (Figure 1f), and significantly more flies than La Costeña ACV or this ACV + 5% apple nectar (F = 19.32, df = 3, 120, *p* < 0.001) independent of sex (F = 0.047; df = 1, 120, *p* = 0.829), or the interaction of attractant*sex (F = 1.180; df = 3, 120, *p* = 0.320). On average, 49 ± 10% (mean ± SD) of captured flies were females across all treatments.

### 3.3. Attraction to Agricultural-Grade ACV in Polytunnel Raspberry Crops

**Figure 2:**
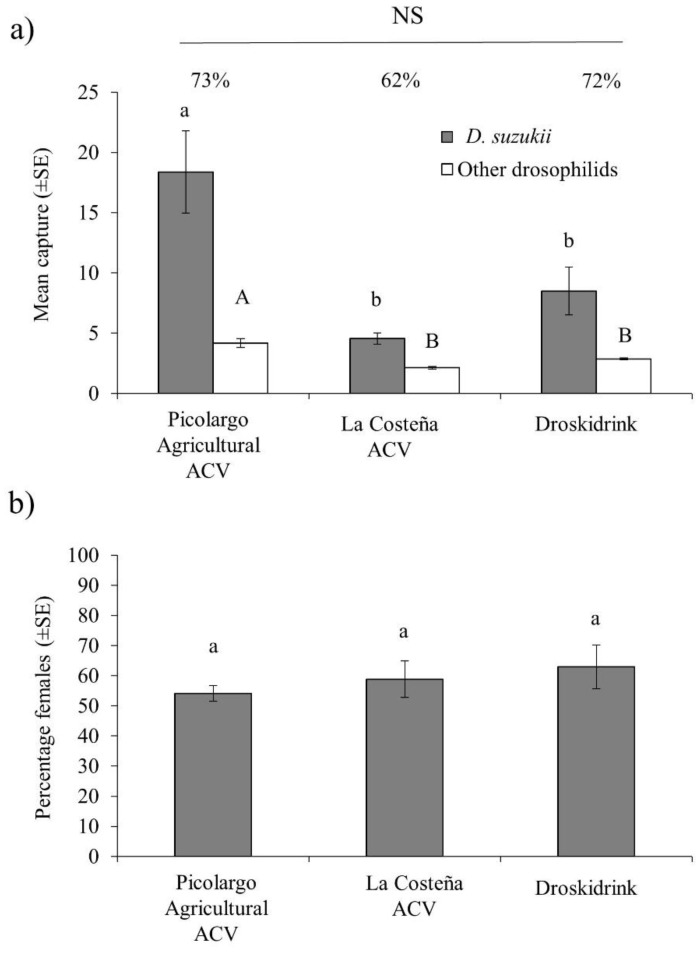
Results of first field trial (**a**) mean number of *D. suzukii* flies and other drosophilids captured per trap per week over the three-week trial and (**b**) mean percentage of females captured in traps containing agricultural-grade ACV, La Costeña ACV and Droskidrink. Columns headed by different letters differ significantly for captures of both sexes of *D. suzukii* (lower case letters) or other drosophilid species (upper case letters) (GLM, *p* < 0.05). Vertical bars indicate SE. Species specificity percentage values are shown above columns in (**a**) (GLM, NS indicates *p* > 0.05).

In the first field trial, a total of 802 drosophilid flies were trapped, with 566 flies (70%) being *D. suzukii*, of which 309 were males and 257 were females. Even though attractants were not renewed during the three weeks of the experiment, agricultural-grade ACV captured a significantly higher number of *D. suzukii* flies than Droskidrink and La Costeña ACV (GLM: χ^2^ = 25.2, df = 2, *p* < 0.001) (Figure 2a). The capture of other drosophilid flies was significantly higher for agricultural-grade ACV (GLM: χ^2^ = 26.2, df = 2, *p* < 0.001), than either Droskidrink or La Costeña ACV (Figure 2a).

Attractant specificity, measured as the ratio of *D. suzukii* to total flies captured per trap, varied between 62–73% among all treatments and did not differ significantly (GLM: χ^2^ = 2.92, df = 2, *p* = 0.232). The percentage of trapped *D. suzukii* females (59–63%) was also statistically similar among the three attractants (GLM: χ^2^ = 1.21, df = 2, *p* = 0.539) (Figure 2b).

**Figure 3:**
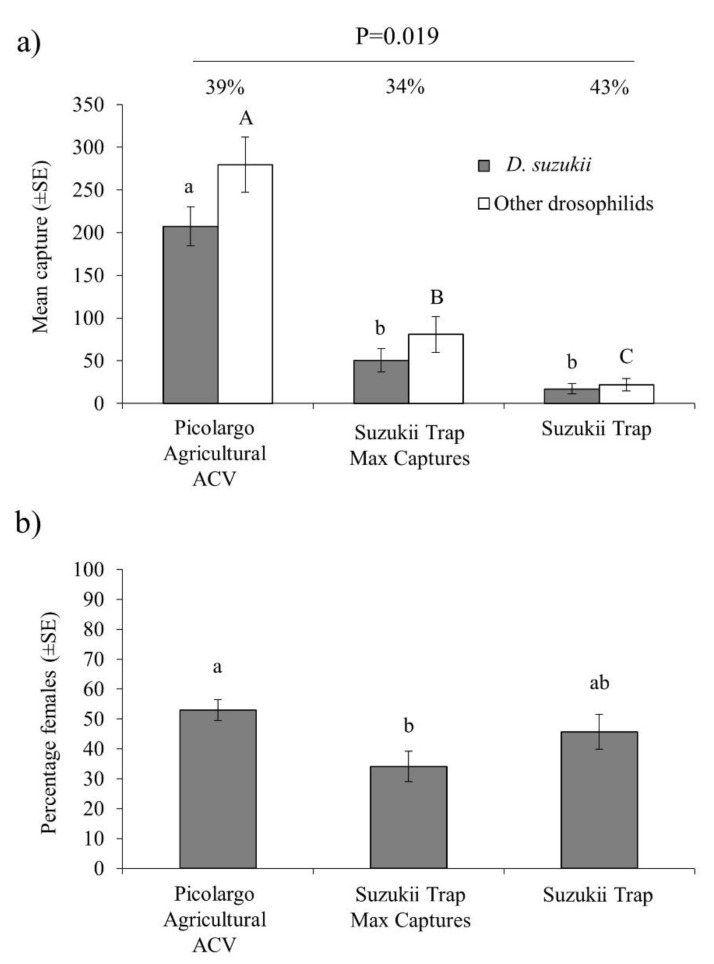
Results of second field trial (**a**) mean number of *D. suzukii* flies and other drosophilids captured per trap per week over the three-week trial and (**b**) mean percentage of females captured in traps containing agricultural-grade ACV, Suzukii Trap and Suzukii Trap Max Captures. Columns headed by different letters differ significantly for captures of both sexes of *D. suzukii* (lower case letters) or other drosophilid species (upper case letters) (GLM, *p* < 0.05). Vertical bars indicate SE. Species specificity percentage values are shown above columns in (**a**) (GLM, *p* = 0.019).

In the second field trial a total of 11,827 drosophilid flies were trapped, of which 4947 flies (42%) were *D. suzukii*, comprising 2895 males and 2052 females. As in the previous trial, agricultural-grade captured between 4-fold and 10-fold more *D. suzukii* flies per trap per week than Suzukii Trap Max Captures or Suzukii Trap, respectably (GLM: χ^2^ = 77.3, df = 2, *p* < 0.001) (Figure 3a). Captures of other drosophilids followed a similar pattern, with the highest captures in traps baited with agricultural-grade ACV, followed by Suzukii Trap Max Captures and Suzukii Trap with the lowest captures of other drosophilids (GLM: χ^2^ = 73.9, df = 2, *p* < 0.001) (Figure 3a).

Attractant specificity, differed significantly among treatments (GLM: χ^2^ = 12.3, df = 2, *p* = 0.002), with the lowest specificity in Suzukii Trap Max Captures (34%), the highest specificity in Suzukii Trap (43%) and intermediate specificity in agricultural-grade ACV (39%) which did not differ significantly from either of the other two attractants. Agricultural-grade ACV captured a significantly higher percentage of females than Suzukii Trap Max Captures, whereas Suzukii Trap captured an intermediate percentage of *D. suzukii* females (GLM: χ^2^ = 7.28, df = 2, *p* = 0.026) (Figure 3b).

## 4. Discussion

We initially observed that La Costeña ACV was as attractive to *D. suzukii* as the commercial attractant Suzukii Trap. A series of caged studies was then performed, comparing mixtures of ACV with different synthetic flavors, hydrolyzed proteins, organic acids and fruit nectars. These tests revealed that the addition of 5% of apple nectar to ACV was the only substance that significantly improved attraction of *D. suzukii*. The addition of acetic acid or propionic acid or dilution of ACV with water (40% diluted ACV) significantly reduced the attraction of *D. suzukii,* whereas mixtures of ACV with hydrolyzed proteins, pineapple and grape nectars, and synthetic fruit essences did not increase the capture of flies over that of ACV alone.

Despite increased captures following the addition of 5% apple nectar to ACV, a higher concentration of 15% apple nectar did not increase fly captures significantly. It seems likely that apple-derived constituents in apple nectar improved the attraction of flies in mixtures with ACV. Chemical analysis of the headspace volatiles of apple juice using GC-MS revealed the presence of highly attractive aromas to *D. suzukii* with clear differences between fresh and fermented juice [29]. A blend of volatile compounds of apple juice (acetic acid, acetoin, ethyl acetate, ethyl octanoate and phenethyl alcohol), produced during fermentation, resulted in at least similar and sometimes higher numbers of *D. suzukii* captured than ACV or other blends of volatile compounds used to bait traps [29].

Fly attraction to red wine vinegar was similar to that of La Costeña ACV in a laboratory cage test, although red wine vinegar was more expensive than ACV, and both vinegars captured significantly more *D. suzukii* flies than sugarcane vinegar or rice vinegar. Previous experiments reported enhanced attractiveness of brown rice vinegar compared with ACV [19,27]. This effect was attributed to the presence of putrescine and spiridine and elevated concentrations of other attractive volatiles, such as acetic acid and acetoin in rice vinegar [18]. However, the brand of rice vinegar used in our test was expensive and its label indicated that it also contained acetic acid, salt, sugar and monosodium glutamate (Table S1).

No marked differences were observed among different brands of ACV, although apple-flavored vinegars (AFV) were significantly less effective than the reference La Costeña ACV. This is likely due to the absence of apple-derived volatiles from apple flavored vinegars that were all composed of sugarcane vinegar or acetic acid mixed with synthetic apple flavors and colorants.

The most striking result of our studies was the very high attraction of *D. suzukii* to Picolargo agricultural-grade ACV that is not fit for human consumption. In laboratory cage studies, this agricultural-grade ACV captured 2- to 3-fold higher numbers of *D. suzukii* than Picolargo human edible grade ACV, La Costeña ACV with or without 5% apple nectar, Suzukii Trap or Suzukii Trap Max Captures, and was equally attractive as Droskidrink (Figure 1d–f). The Droskidrink combination of ACV, red wine and sugar has been hailed as an effective food bait developed in Europe to monitor *D. suzukii* and was demonstrated to capture higher numbers of *D. suzukii* than a standard ACV under cage and polytunnel tests [13,26,38]. However, Picolargo agricultural-grade ACV clearly outperformed Droskidrink in the polytunnel trail performed by us in raspberry (Figure 2a), and also outperformed both Suzukii Trap formulations in the second field trail (Figure 3a).

ACV is relatively stable and berry growers in Mexico usually renew ACV-baited traps at intervals of 2–3 weeks. The commercial formulations of Suzukii Trap are also designed to be changed at intervals of several weeks. Although we did not investigate possible ageing effects in any of the attractants tested, the captures over the three week period reflect the performance of attractants under current berry production practices in Mexico. In a previous study, captures of *D. suzukii* did not change significantly between 7-day-old and 14-day-old ACV, although the number of insects captured was generally low [21]. Similarly, we did not observe marked reductions in the numbers of flies captured in weekly samples during field experiments (data not shown), suggesting that none of the attractants tested lost attractiveness during the polytunnel trails.

Field trials revealed remarkably similar percentages of females captured among the different attractants which varied around 50–60% females, except for Suzukii Trap Max Captures that captured a significantly lower percentage of females (34%) (Figure 3b). Previous studies have observed the capture of a similar percentage of females between ACV and Suzukii Trap [22].

The abundance of *D. suzukii* in both trials was related to the crop management and phenology. The first experiment was performed during the peak production period, whereas the second experiment was carried out at the end of crop season when fallen damaged fruits were not being removed from the ground, allowing other drosophilid species to exploit these fruits and increase their populations.

Although the abundance of other drosophilids varied according to crop management practices, 72% and 42% of flies captured by agricultural-grade ACV traps were *D. suzukii* in the first and second polytunnel trials, respectively. In both trials, agricultural-grade ACV had a similar specificity to that of Suzukii Trap formulations or Droskidrink. Suzukii Trap has been described as highly selective for *D. suzukii* in previous evaluations [22,25,26], but the results were similar to Droskidrink in terms of specificity in some experiments [26]. High variation in specificity of different attractants is often reported, and *D. suzukii* often comprises less than one-third of the total capture of drosophilid flies in studies on traps baited with ACV-based attractants [10,11,21].

A recent study has indicated that the attraction of *D. suzukii* to ACV varies seasonally in Europe, with reduced attraction in the summer months when more fruits are available [17]. Attraction to fermentation cues over fruit-based cues depended on nutritional, reproductive and mating status, and was strongly influenced by temperature. Indeed, a summer period of low captures has been reported in several European countries [14,15,16], whereas different seasonal capture patterns have been observed in California [42]. Certain researchers have also reported that *D. suzukii* responses to ACV and other attractants may not accurately predict the true incidence of pest populations, forcing growers to adopt a calendar-based schedule of insecticide applications, irrespective of trap captures, as a risk-hedging strategy in the face of uncertain pest pressure [32,33]. To date, variation in *D. suzukii* attraction to ACV has not been observed in Mexico, although the possibility that Mexican populations of this pest experience a seasonal shift in olfactory preferences, such as that observed in Europe [17], deserves systematic study.

The chemical factors that promote the notable response of *D. suzukii* to agricultural-grade ACV merit further investigation. This was clearly an unpasteurized or unsterilized, and mostly unfiltered product that had a cloudy appearance, from which sediment accumulated if allowed to stand for several hours. It seems likely that the sediment was derived from apple debris and fermentation products that are invariably removed from high-grade vinegars for human consumption. The agricultural-grade ACV did not contain specific preservatives or stabilizers that are included when ACV is formulated for human consumption (Supplementary Material Table S1). Vinegars are also formulated with additives to prevent browning, haze formation and bacterial growth. Approved additives include compounds such as sulfur dioxide, sodium benzoate and sodium metabisulfite, among others [43]. The likely presence of acetic acid bacteria in the agricultural-grade product could have improved the fly’s response as volatiles from these bacteria are attractive to *D. suzukii* [44].

Based on their cost in supermarket outlets in Mexico, there was not great variation in the price of different ACV brands (USD 0.8–0.9/L), with the exception of the organic Heinz ACV that was 2.5-fold more expensive, and Ruiz ACV that cost almost half as much as the other ACVs. Picolargo agricultural ACV was only 5% cheaper than the edible grade ACVs, but cost 7-fold less than Suzukii Trap and 2.5-fold less than Droskidrink.

## 5. Conclusions

We conclude that ACV products are highly attractive to *D. suzukii* and captures can be increased by the addition of 5% apple nectar. Mixtures of ACV with products containing hydrolyzed protein did not increase captures, whereas synthetic fruit aromas and apple flavored vinegars performed poorly. An agricultural grade ACV, unfit for human consumption, captured significantly more *D. suzukii* flies in field trials than two formulations of Suzukii Trap or the ACV, wine and sugar mixture known as Droskidrink. The high attractiveness and low cost of agricultural-grade ACV makes it a useful tool for monitoring *D. suzukii* populations in berry crops. The degree to which these characteristics favor the potential use of this product for mass trapping of this pest could be established in future studies.

## Figures and Tables

**Table 1:** Mean numbers of *D. suzukii* flies captured in the La Costeña apple cider vinegar (ACV) reference treatment used in all tests compared to captures of flies in cup traps baited with Suzukii Trap (Experiment 1) or ACV that had been modified by the addition of other substances (Experiments 2–6).

Experiment	Treatments	Mean Capture ± SE	Sig.	Statistical Values
Exp. 1	ACV	19.4 ± 2.0	NS	t = 0.357, df = 7, *p* = 0.731
	Suzukii Trap	18.1 ± 1.8		
Exp. 2	ACV	17.8 ± 3.3	NS	t = 0.326, df = 7, *p* = 0.754
	ACV 80%	15.9 ± 2.7		
	ACV	18.9 ± 2.1	NS	t = 1.48, df = 7, *p* = 0.181
	ACV 60%	12.8 ± 2.4		
	ACV	26.3 ± 1.0	***	t = 5.57, df = 7, *p* < 0.001
	ACV 40%	10.3 ± 2.0		
Exp. 3	ACV	24.9 ± 2.5	***	t = 7.72, df = 7, *p* < 0.001
	ACV + 5% acetic acid	9.0 ± 2.0		
	ACV	21.6 ± 1.8	***	t = 8.75, df = 7, *p* < 0.001
	ACV + 2.5% propionic acid	3.4 ± 0.5		
Exp. 4	ACV	15.6 ± 2.3	NS	t = 0.683, df = 7, *p* = 0.516
	ACV + 8% Captor	17.9 ± 1.7		
	ACV	17.3 ± 1.8	NS	t = 0.710, df = 7, *p* = 0.501
	ACV + 8% Winner	14.8 ± 2.2		
	ACV	20.6 ± 2.3	NS	t = 2.06, df = 7, *p* = 0.079
	ACV + 8% Flyral	13.6 ± 2.7		
	ACV	15.1 ± 1.2	NS	t = 0.848, df = 7, *p* = 0.425
	ACV + 8% CeraTrap	13.5 ± 1.2		
Exp. 5	ACV	15.8 ± 1.8	NS	t = 0.738, df = 7, *p* = 0.485
	ACV + 0.1% raspberry flavor	15.9 ± 1.9		
	ACV	17.6 ± 2.7	NS	t = 0.504, df = 7, *p* = 0.630
	ACV + 0.1% blackberry flavor	12.5 ± 1.6		
	ACV	13.8 ± 2.2	NS	t = 0.895, df = 7, *p* = 0.400
	ACV + 0.1% strawberry flavor	17.3 ± 2.3		
	ACV	16.6 ± 1.2	NS	t = 1.24, df = 7, *p* = 0.225
	ACV + 0.1% apple flavor	14.9 ± 1.6		
Exp. 6	ACV	19.1 ± 2.8	NS	t = 1.01, df = 7, *p* = 0.344
	ACV + 5% grape nectar	15.1 ± 1.5		
	ACV	13.5 ± 1.4	NS	t = 0.654, df = 7, *p* = 0.606
	ACV + 5% pineapple nectar	12.3 ± 1.2		
	ACV	11.1 ± 1.0	***	t = 5.165, df = 7, *p* = 0.001
	ACV + 5% apple nectar	21.8 ± 1.4		
	ACV	14.9 ± 1.3	NS	t = 1.50, df = 7, *p* = 0.178
	ACV + 15% apple nectar	20.1 ± 2.3		

All results were subjected to paired *t*-test. Sig. indicates significance; NS indicates *p* > 0.05; *** indicates *p* < 0.001.

## Supplementary Materials

The following are available online at https://www.mdpi.com/2075-4450/11/7/448/s1, Table S1: Characteristics of different brands of vinegars used in the study.
