# Supplementary material for: Agricultural-Grade Apple Cider Vinegar Is Remarkably Attractive to Drosophila suzukii (Diptera: Drosophiliadae) in Mexico
Source: Insects. 2020 Jul 15;11(7):448. doi: 10.3390/insects11070448 (PMC7411812; doi:10.3390/insects11070448)
Supplement: Supplementary file 1 [file insects-11-00448-s001.pdf]

Table S1. Characteristics of different brands of vinegars used in the study

Vinegar type	Brand	Acidity	Label composition	Stabilizer/Preservative
ACV	La Costeña	5%	Apple cider vinegar diluted in water to 5% acidity with caramel color	Sodium metabisulfite
ACV	Clemente Jacques	5%	Apple cider vinegar plus sugarcane vinegar diluted in water to 5% acidity	Sodium metabisulfite
Rice vinegar	Sugoi	undefined	Acetic acid, sugar, salt, water and monosodium glutamate	Sodium metabisulfite
Red wine vinegar	Carbonell	6%	Red wine vinegar	Sulfur dioxide
ACV	Clemente Jacques	5%	Apple cider vinegar diluted with water, sugarcane vinegar with synthetic apple flavor and caramel color	Sodium metabisulfite
ACV	Heinz	5%	Ultra-filtered organic apple cider vinegar diluted to 5% acidity.	undefined
ACV	Barrilito	5%	Apple cider vinegar diluted in water to 5% acidity	undefined
ACV	Ruiz	undefined	Undefined	undefined
ACV	Picolargo edible grade	5%	Meets regulations NMX-F-122-1968, CODEX-STAN-162-1987 for human consumption	undefined
ACV	Picolargo agricultural	5%	For use as an organic herbicide and for cleaning agricultural irrigation equipment	none
AFV	La Faburrita	5%	Unspecified vinegar, water, apple concentrate and caramel color	Sodium benzoate
AFV	San Miguel	5%	Unspecified vinegar diluted to 5% acidity with natural colorant	undefined
AFV	La Anita	undefined	Acetic acid, water, synthetic apple flavor and a mixture of red, blue, yellow and tartrazine colorants	undefined

ACV = apple cider vinegar

AFV = apple flavored vinegar
